# What can we learn from the SARS-COV-2 pandemic about the value of specific radiological examinations?

**DOI:** 10.1186/s12913-021-07190-w

**Published:** 2021-10-26

**Authors:** Bjørn Hofmann, Eivind Richter Andersen, Elin Kjelle

**Affiliations:** 1 Institute for the Health Sciences at the Norwegian University of Science and Technology (NTNU) at Gjøvik, PO Box 191, N-2802 Gjøvik, Norway; 2 Centre of Medical Ethics at the University of Oslo, Oslo, Norway

**Keywords:** Low-value care, Appropriateness, Choosing wisely, Ethics, Covid-19, SARS-COV-2

## Abstract

**Background:**

The SARS-COV-2 pandemic provides a natural intervention to assess practical priority setting and internal evaluation of specific health services, such as radiological services. Norway makes an excellent case as it had a very low infection rate and very few cases of COVID-19. Accordingly, the objective of this study is to use the changes in performed outpatient radiological examinations during the first stages of the SARS-COV-2 pandemic to assess the practical evaluation of specific radiological examinations in Norway.

**Methods:**

Data was collected retrospectively from the Norwegian Health Economics Administration (HELFO) in the years 2015–2020. Data included the number of performed outpatient imaging examinations at public hospitals and private imaging centers in Norway and was divided in to three periods based on the level of restrictions on elective health services. Results were analyzed with descriptive statistics.

**Results:**

In the first period there was a 45% reduction in outpatient radiology compared to the same time period in 2015–2019 while in period 2 and 3 there was a 25 and 6% reduction respectively. The study identified a list of specific potential low-value radiological examinations. While some of these are covered by the Choosing Wisely campaign, others are not.

**Conclusion:**

By studying the priority setting practice during the initial phases of the pandemic this study identifies a set of potential low value radiological examinations during the initial phases of the SARS-COV-2 pandemic. These examinations are candidates for closer assessments for health services quality improvement.

## Key points

- The SARS-COV-2 pandemic has reduced the outpatient radiological examinations with 45, 25 and 6% during the three initial phases of the pandemic.
- The pandemic provides a natural intervention to study the value of specific radiological examinations.
- A set of specific examinations are identified as candidates for health services quality improvement.

## Introduction

**Fig. 1:**
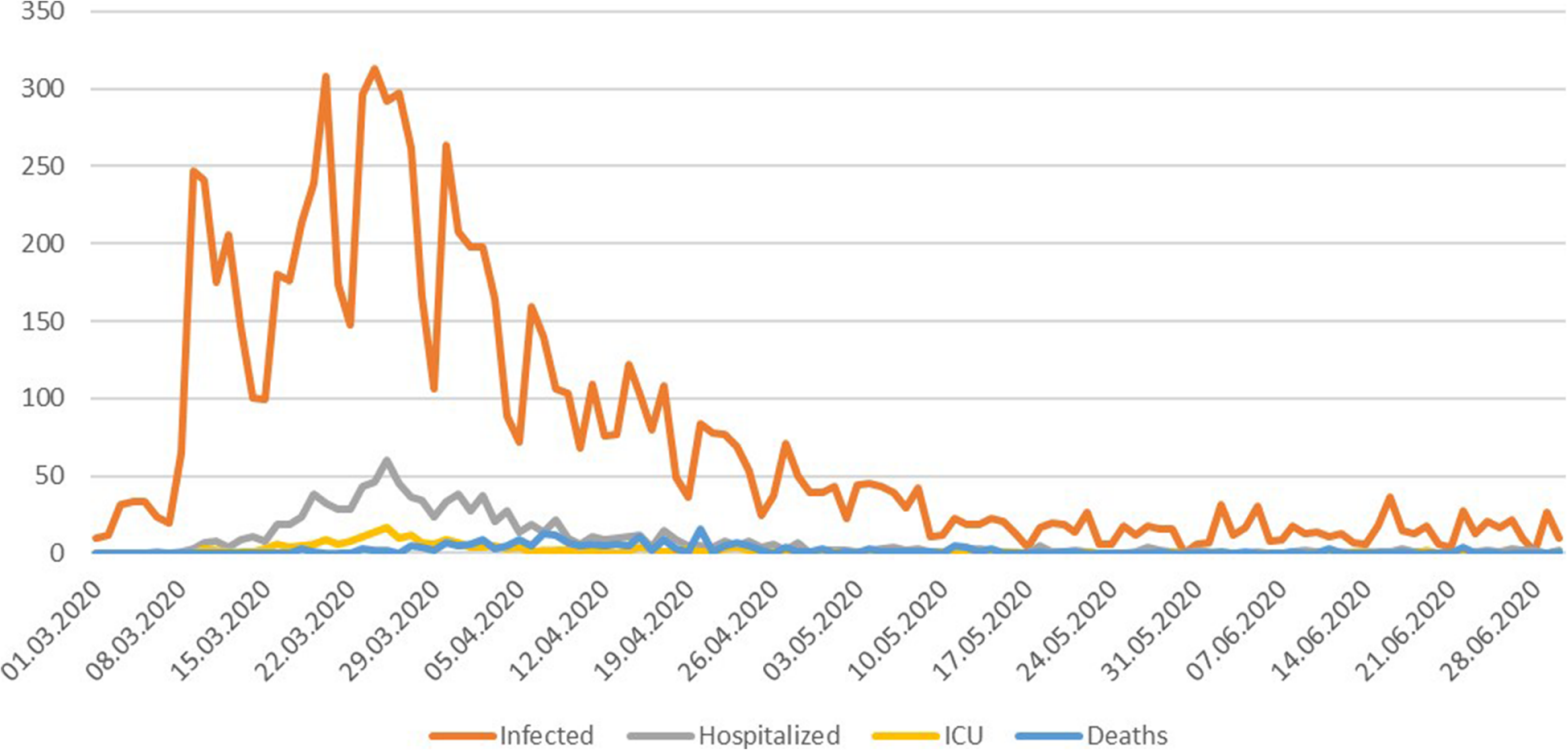
The number of tested (positive), infected, hospitalized, persons in the ICU, and deaths during the first months of the outbreak in Norway from March 1 to June 30. Data are available at the Norwegian Institute of Public health (www.fhi.no)

During the first period of the SARS-COV-2 pandemic in Norway (March–June 2020), activities at Norwegian hospitals were reduced to a minimum, including postponing outpatient services, elective surgery, and scheduled follow ups. However, the number of persons affected by the pandemic was very small compared to other countries. Figure 1 shows some key figures for the first months of the outbreak in Norway. By June 30 there were 251 covid-19 related deaths in Norway.

While very few health services were directly involved in handling covid-19 patients, all were significantly affected as elective activities were initially reduced to a minimum and then gradually opened again. The event provides a natural intervention to study health services practical priority setting and an unprecedented opportunity to assess practical evaluation of specific health services [1, 2].

One specific area for investigation is radiological services. Radiological technologies provide tremendous opportunities for diagnostics and subsequent treatment and care. This has significantly expanded the possibilities to help people [3], but also the potential for unnecessary, inappropriate, futile, or even harmful examinations [4–6]. Furthermore, critical reflections have emerged on whether there is “too much medicine” [7–12] and too much radiology [4, 13–16].

Internationally a wide range of campaigns, such as Choosing Wisely, Too Much Medicine (BMJ), Smarter Medicine, Prudent Health Care, Slow Medicine, Do Not Do (NICE) [17], have focused on (in)appropriate and low value care, defined as “*an intervention in which evidence suggest it confers not or very little benefit for patients, or risk of harm exceeds probable benefit or, more broadly, the added costs of the intervention do not provide proportional added benefits*” [18]. Accordingly, a negative test can have positive value and a positive test can have low value. While there are many consensus-based suggestions for low-value radiological services [5, 19–22] the extension of low value radiology is still unclear, as examinations identified as “low-value” can be of great value in specific cases [23]. Hence, it can be difficult to define and identify low-value care in radiological practice. This is especially important in perspective of value-based radiology [24–26].

However, the pandemic provides a unique opportunity to investigate what happens to health services when forced to prioritize in a strict manner. From an ethical perspective, one should always try to learn as much as possible when crisis occurs.

Thus, the purpose of this study was to assess changes in performed outpatient radiological examinations during the first stages of the SARS-COV-2 pandemic and apply this to study the practical evaluation of radiological examinations. In order to improve health services, we focus on examinations that potentially are of low value.

To address this overall issue the specific research questions are:

- Which examinations were reduced most during the pandemic?
- What are the patterns of reduced radiological services during the SARS-COV-2 pandemic in 2020?
- How well does the reduction in services correspond to the recommendations of the Choosing Wisely Campaign in Norway and the USA?

## Material and methods

The data for this study was all outpatient radiological examinations registered at the Norwegian Health Economics Administration (HELFO) for three specific periods in the years 2015–2020. The periods were defined by the reactions of the Norwegian health authorities and the Government to the SARS-COV-2 pandemic in 2020:

*Period 1* is from March 12 till April 11, “the shut-down period”, during which only extraordinarily important and severe cases were examined. Several outpatient services were shut down and elective surgery was postponed.

*Period 2* from April 12 till May 12, a slight let up in restrictions and important cases were examined in addition to increased outpatient activity in general.

*Period 3* is from May 13 till June 12, further let up in restrictions and the radiology departments returned to almost normal outpatient activities.

Results were analyzed with descriptive statistics in Microsoft Excel for Office 365 ProPlus.

Data were grouped and subsumed under main codes (2020) including additional codes from the Norwegian Classification of Radiological Procedures (NCRP) [27]. Codes from 2015 were transposed to 2020 version of codes, as there was a major shift in nomenclature from 2015 to 2016. The average and relative standard deviation was calculated for each code for each period and compared to the subsumed number of examinations for the corresponding month in 2020.

**Fig. 2:**
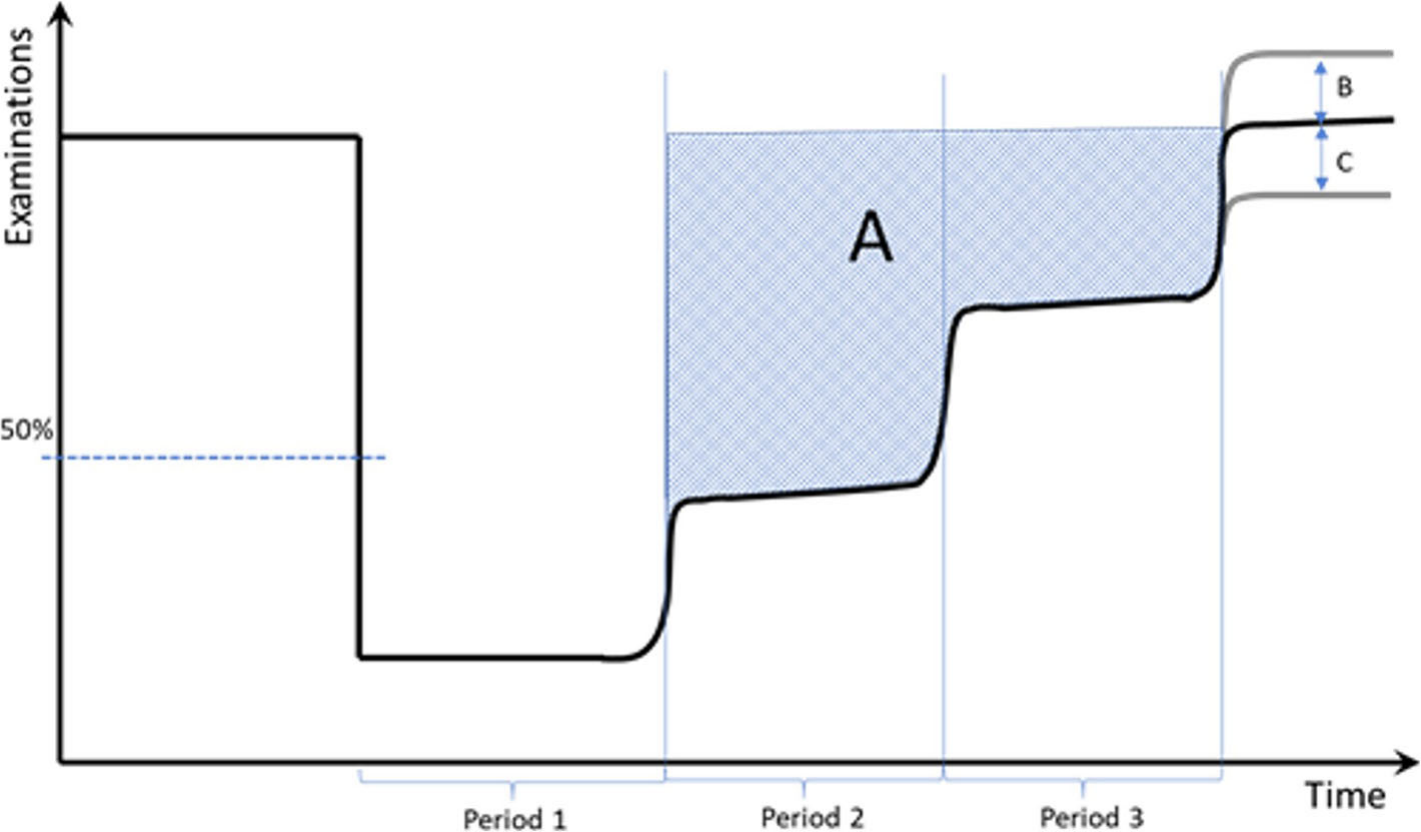
Model used for measuring reduced examinations during the various periods of the pandemic. A refers to the total volume of examinations reduced during the phases when the radiological departments were opening up. B indicates an increased activity post opening and may indicate a backlog while C indicates a long-lasting reduction

Changes less than 50% for specific examinations in Period 1 were excluded as these examinations were considered to be of high value. Figure 2 illustrates the model applied in this study. In order to avoid artifacts due to small numbers, averages of less than 100 examinations per months were excluded. Reduction in the number of examinations was calculated as the difference in a given period (Period 1–3) in 2020 from the stable average for the same period for the years 2015–2019 in percent.

## Results

There were about 256,000 examinations for all three periods between 2015 and 2019 and the relative standard deviation varied between 4 and 12%, as can be seen in Table 1. The reduction for each period from 2015 to 2019 to 2020 varies from 45.5% in Period 1 to 6.1% in Period 3.

**Table 1:** Average outpatient main examinations for three periods in 2015–2019 compared to the same periods in 2020

	Average 2015–2019	SD	%RSD	2020	Overall reduction (%)
Period 1	254,424	29,391	11.6	140,328	45.5
Period 2	256,695	24,859	9.7	194,375	25.4
Period 3	259,724	10,281	4	247,366	6.1

Table 2 shows the reduction in number of examinations for the four main radiological modalities from 2019 to 2020 for each of the three periods. While the number of examinations were reduced for all modalities during the first period, the lowest relative reduction was in ultrasound. The use of ultrasound increased during the second period and then decreased again while CT increased in the last period compared to 2019.

**Table 2:** Reduced examinations for different modalities in all three periods comparing 2019 and 2020 in numbers and percentages. Negative reduction means increase

Examination	Period 1		Period 2		Period 3	
	Reduction (n)	Reduction (%)	Reduction (n)	Reduction (%)	Reduction (n)	Reduction (%)
CT	33,803	57.57	15,600	33.48	−12,374	−20.87
Conventional radiography	59,094	57.52	8474	12.84	8943	11.56
MRI	48,833	56.17	9142	15.27	12,338	16.35
Ultrasound	16,614	36.11	−11,444	−28.8	9516	17.2

**Fig. 3:**
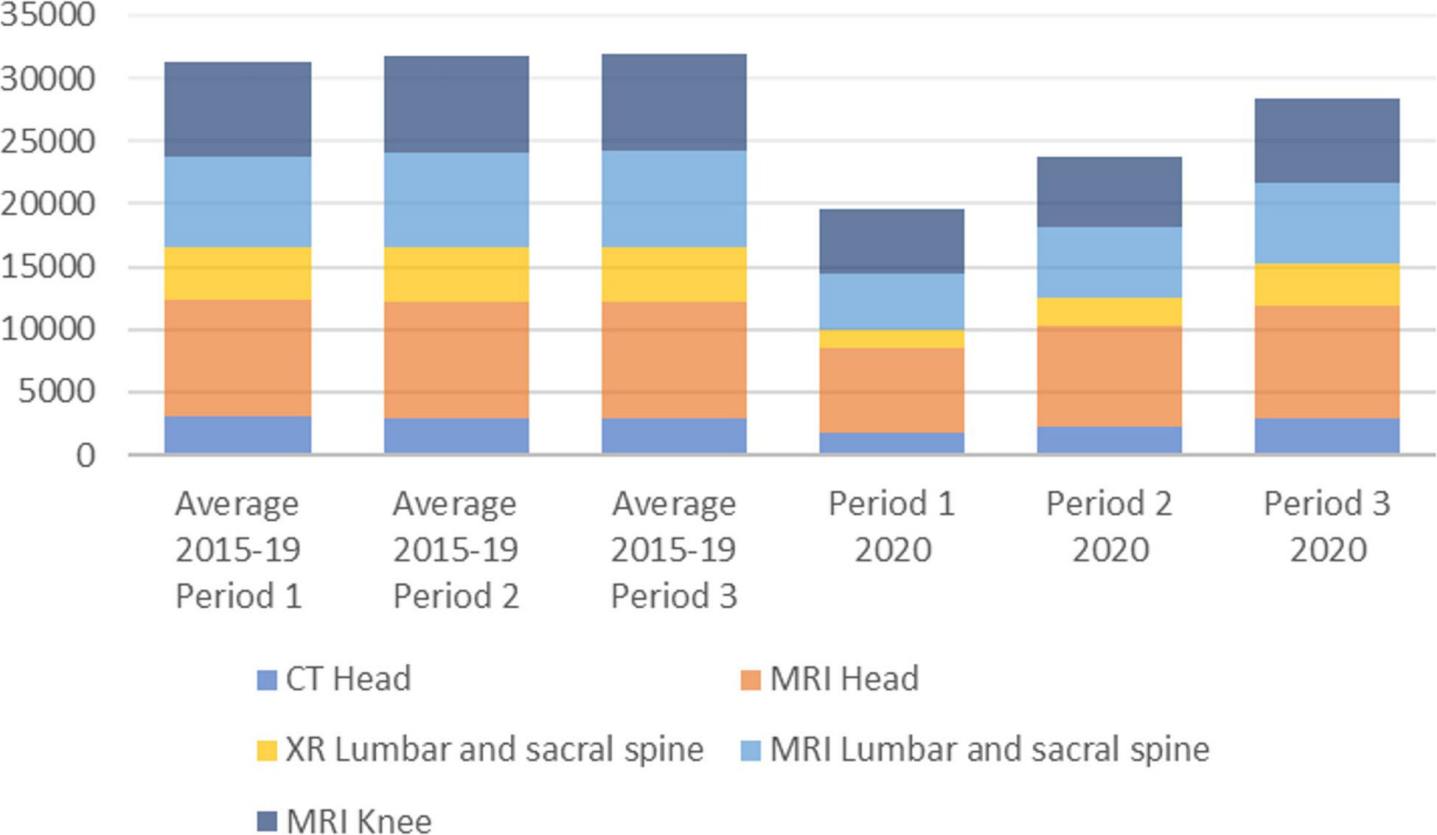
The average number of examinations for periods P1-P3 for the years 2015–2019 compared with the number of examinations for the same periods for 2020 for codes related to examinations in the Norwegian Choosing Wisely campaign

For the main examinations in adults identified by the Norwegian version of the Choosing Wisely Campaign [28], the development of the number of examinations are shown in Fig. 3.

As the figure illustrates, most of the identified examinations were reduced from one to two third of the normal level but increasing in period 2 and 3.

**Fig. 4:**
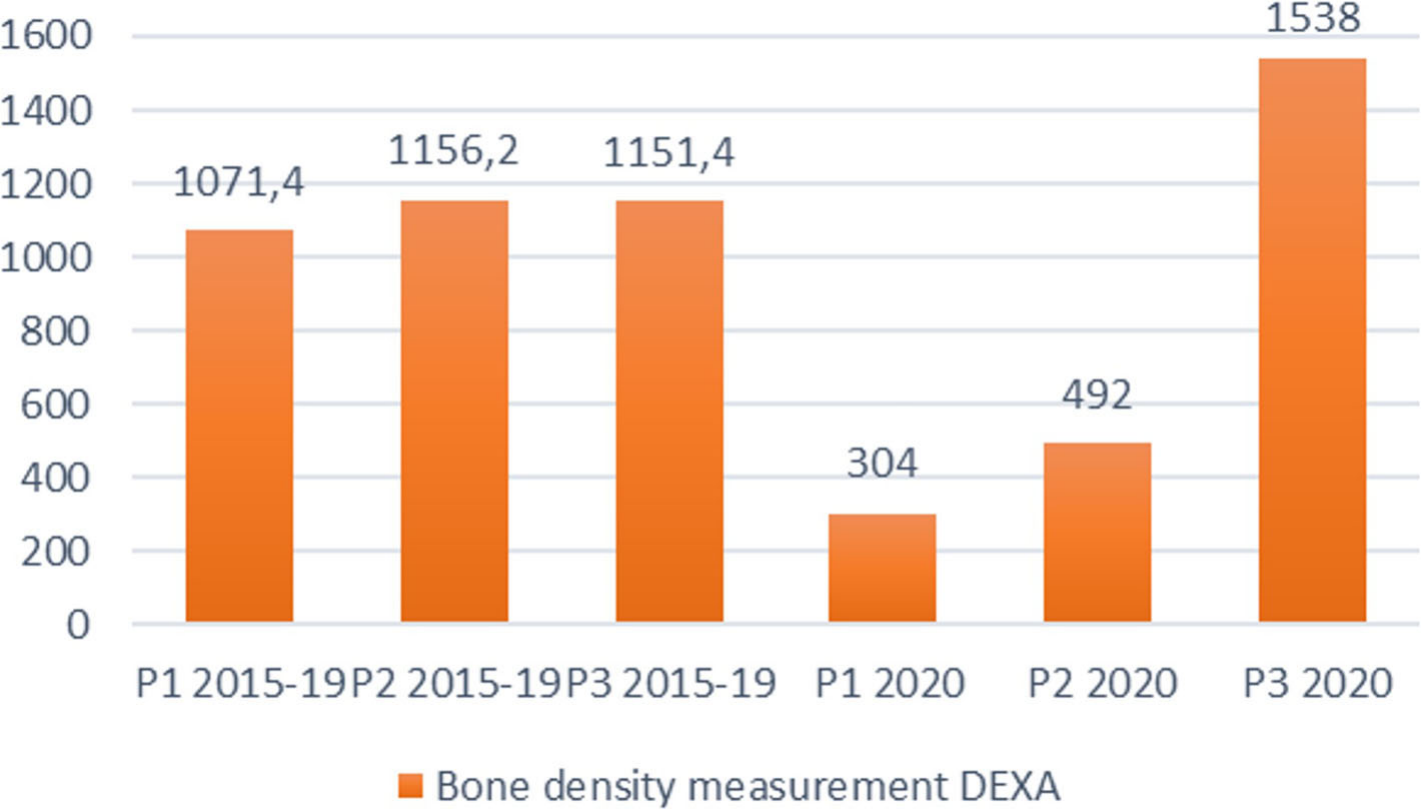
The average number of examinations for bone density measurement (DEXA) for the periods (P) 1–3 for the years 2015–2019 compared with the number of examinations for the same periods for 2020

While the use of most examinations followed the pattern in Table 1 and Fig. 2, i.e., with a stable number of examinations for all three periods (2015–2019), and a substantial reduction in the first period and then a gradual increase in the  subsequent periods in 2020, some examinations had a significant reduction in period 1 however increased beyond the previous average in period 3. One example of this is bone density measurement (DEXA), illustrated in Fig. 4.

**Fig. 5:**
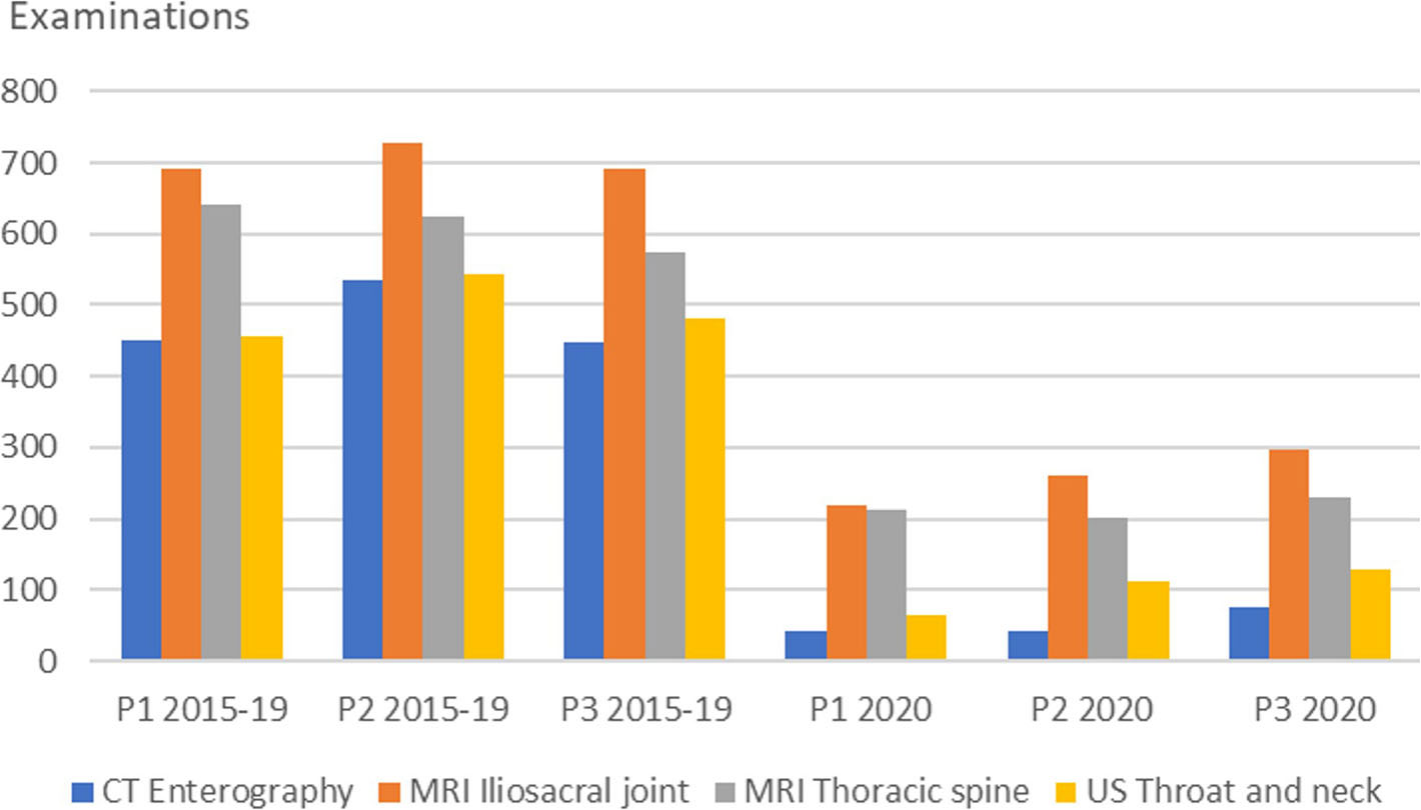
Examinations for selected examinations where the number of examinations stayed low for all three periods (P) in 2020

Further, some types of examinations were substantially reduced during all three periods as shown in Fig. 5.

**Fig. 6:**
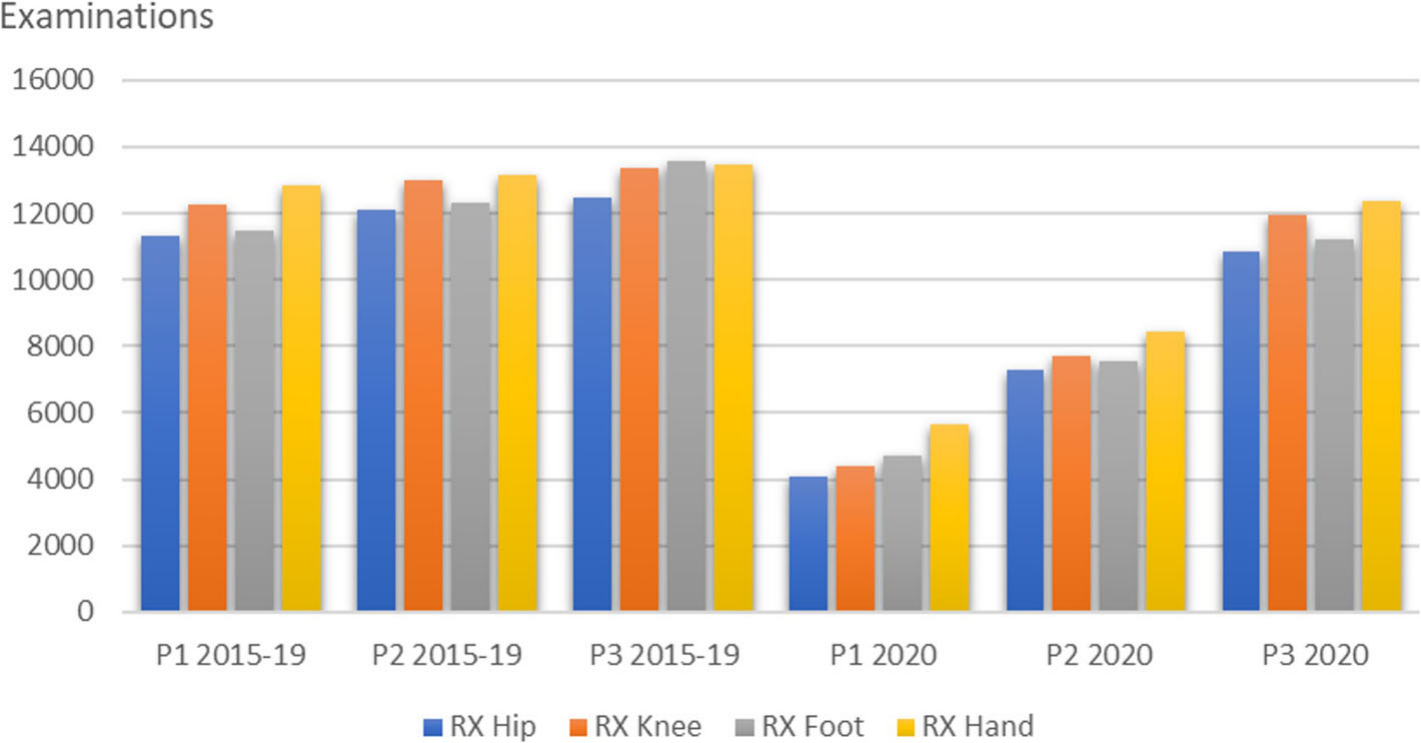
Examples of examinations where the initial reduction was reduced from Period (P) 1 to Period 3

Other examinations had a larger reduction during Period 1, however still regained activity during Period 2 and 3. Examples of this are typical conventional x-ray examinations of the hip, knee, foot and hand, as shown in Fig. 6, which reveals a small expected seasonal increase for these examinations.

Table 3 shows the examinations that had a large reduction (> 20%) in period 2 and durable reduction (> 10%) on average for period 2 and 3. The number of examinations is given to indicate the volume. As the table demonstrates, a series of examination have more than 50% reduction even in period 2 and 3: KUB view, CT Enterography, MRI Pelvis and lower limb, Throat and neck ultrasound, MRI Thoracic spine, MRI sacroiliac joints, Liver, gall bladder, and pancreatic ultrasound, Axillary ultrasound and MRI of the face. CT was the modality with the lowest number of examination codes to have a large reduction in use, while ultrasound and conventional radiography had the largest amount of examination codes with a large reduction in use. Among the defined low value examinations in the Choosing Wisely campaign, imaging of the spine [28] and ultrasound of the throat and neck [5] had the largest reduction.

**Table 3:** Average reduction (in %) for period (P) 2 and 3 for examinations with more than 20% reduction for period 2 including the average number of examinations for each period 2015–2019 as well as for 2020

Examination	Average reduction in P2 and P3 (%)	Exam. in P1 2015–2019	Exam. in P2 2015–2019	Exam. in P3 2015–2019	Exam. in P1 2020	Exam. in P2 2020	Exam. In P3 2020
**Conventional Radiography**							
KUB (Kidney, ureters and bladder)	86.8	107	98	101	20	12	11
Orthopantomography	47.5	168	174	167	10	78	80
Sacroiliac joints ^ab^	47.2	299	310	326	74	112	184
Cervical spine	43.2	1417	1407	1319	466	610	816
Sacrum and coccyx	36.8	297	275	234	74	138	172
Chest ^b^	30.4	23,868	23,265	22,715	9557	13,215	16,856
Thoracic, lumbar, and sacral spine ^b^	27.9	549	532	544	185	256	468
Abdominal	27.5	754	738	761	330	418	597
Lumbar and sacral spine^ab^	26.8	4180	4244	4281	1382	2307	3440
Thoracic and lumbar spine ^b^	26.5	232	271	233	76	151	183
Thoracic spine ^b^	25.9	1184	1166	1111	446	684	909
Ribs	22.4	297	285	265	124	185	226
Shoulder	22.2	6901	6452	6118	2890	4140	5340
Calf	19.6	2312	2261	2157	1092	1532	1877
Foot ^b^	19.6	11,463	12,315	13,551	4695	7565	11,211
Hip	19.0	11,291	12,101	12,462	4085	7300	10,825
Knee	18.4	12,262	13,002	13,347	4402	7721	11,967
Total spine	16.5	714	733	737	203	494	644
Hand	16.3	12,825	13,138	13,448	5638	8437	12,360
Ankle ^b^	13.8	8765	9262	9520	4226	6470	8573
Upper arm	13.3	964	892	859	569	676	832
Pelvis	13.2	8626	8954	9164	3348	5960	8677
Wrist	12.7	9424	8903	9203	4892	6405	8902
Clavicle	12.1	1352	1295	1395	642	983	1274
Femur	11.7	814	796	791	376	599	747
**Computed tomography**							
Enterography	85.4	451	535	448	42	42	77
Colonography with fecal tagging	19.5	437	488	496	295	359	412
Face ^b^	17.6	473	477	488	230	326	422
Sinuses ^b^	15.5	2670	2542	2358	1392	1878	2187
Lumbar and sacral spine^ab^	13.8	416	432	435	204	311	390
**Magnetic resonance imaging**							
Pelvis and lower limb	81.1	207	263	261	22	35	44
Thoracic spine ^b^	60.4	642	626	575	212	203	231
Sacroiliac joints ^b^	55.7	693	729	691	218	260	298
Face ^b^	49.6	265	263	249	83	121	114
Cervical spine ^b^	27.5	3513	3513	3362	1970	2224	2502
Head and MRA Brain ^b^	18.1	171	187	181	96	132	144
Lumbar and sacral spine^ab^	17.4	7285	7620	7725	4589	5491	6401
Hip	16.6	2034	2029	2028	1317	1429	1816
Total spine ^b^	16.5	686	743	733	432	529	621
Knee^ab^	14.3	7433	7560	7707	5047	5713	6766
Calf	13.3	362	363	387	282	287	336
Pelvis ^b^	13.2	2301	2332	2283	1614	1752	2112
**Ultrasound**							
Throat and neck ^b^	71.9	457	542	482	65	112	129
Liver, Gall Bladder, and Pancreas	53.3	610	721	652	114	198	351
Axillar	52.7	615	782	640	171	256	318
Pelvis	45.4	109	104	106	28	45	60
Breast	43.0	1795	1953	1812	723	905	1024
Abdomen and pelvis ^b^	41.5	3540	3519	3331	813	1500	2165
Fine needle aspiration cytology of the breast	40.0	482	505	502	222	271	277
Liver Ultrasound Elastography	38.0	96	111	97	20	47	67
Abdominal aorta	30.6	587	658	635	161	312	487
Fine needle aspiration cytology of thyroid	29.0	198	186	210	80	113	148
Carotid arteries	27.5	150	155	149	35	93	109
Scrotum	27.5	1600	1620	1535	577	921	1212
Kidneys	26.4	520	571	553	203	344	406
Thyroid ^b^	26.0	838	869	848	187	450	712
Thigh	25.1	109	122	106	30	59	97
Knee	24.7	180	183	189	89	120	140
Urinary tract	24.6	1304	1352	1257	429	771	1057
Liver	22.4	505	536	534	161	307	451
Abdomen	21.5	1947	1937	1803	790	1178	1613
Skin and subcutaneous	20.7	341	316	311	107	194	284
Calf	16.8	133	135	149	48	91	126
Bladder ultrasound with Post-void residual volume measure	16.7	142	140	140	66	73	146
Lower limb veins ^b^	12.8	925	970	1013	536	731	886

^a^Codes related to the Choosing Wisely Campaign in Norway [28], ^b^ Codes related to the Choosing Wisely Campaign in the USA [5]

## Discussion

This study shows a substantial reduction of the number of outpatient examinations during the initial phases of the SARS-COV-2 pandemic in 2020 and concur with other studies [29–34]. As a natural intervention it can teach us about practical priority setting, i.e., how specific radiological examinations are valued in practice. As such, it can help us identify potential low value radiological services.

Clearly, low value care cannot be read out of the data directly. Careful scrutiny of examination codes and indications is necessary. Moreover, we must assess whether there are unintended consequences associated with the abrupt reduction in imaging, e.g., delayed diagnoses and treatments, and excess mortality. For example, it has been documented that the number of treated injuries was reduced during the first weeks of the pandemic [35], that the reduction in imaging exacerbated inequities [36], had economic consequences [37], and influenced radiology trainees [38]. However, it is too early to assess the implications of the reduction in imaging on people’s health. It is also important to notice that health services in general were mostly reduced for milder illnesses [39]. Hence, the study is an important step in identifying low-value care and to improve the health services. The approach demonstrates how we can learn from the pandemic and it supplements other ways to identify low value care. The approach is also recognized and applied in many other fields [39–48] to identify and reduce a range of low-value services.

The reduction in the first period cannot be used to assess the value of radiological services as it certainly included reduction of high value services. However, reduction of examinations during the second and third period, can teach us about priority setting in practice. Accordingly, the following examinations are candidates for being of low value and merit further investigation: Abdominal KUB view, CT Enterography, MRI Pelvis and lower limb, Throat and neck ultrasound, MRI Thoracic spine, MRI sacroiliac joints, Liver, gall bladder and pancreatic ultrasound, Axillary ultrasound and MRI of the face. Of these, Throat and neck ultrasound, MRI of Thoracic spine, sacroiliac joints and face are the ones related to Choosing Wisely recommendations [5].

Our study provides useful insights of the practical priority setting of radiological services. Interestingly, we found that utilization of bone density examinations (DEXA), increased in the third period after the lockdown. This can be the result of a backlog, but also because this service is provided by special departments with few other services and high capacity. However, frequent DEXA screening for osteoporosis in elderly is in the literature considered as low value care [49]. This underscores our point that the identified examinations need further scrutiny.

Only five CT-examinations were reduced more than 20% in period 2 (Table 3), despite a 57% reduction in the first period. The high utilization of CT during the pandemic may be due to prioritizing cancer pathways and cancer follow up, which was prioritized in the Norwegian health services during the lock down [50]. This could indicate that most CT-examinations are of high value or that it is difficult to reduce the use of CT examinations in Norwegian hospitals. This merits further scrutiny since CT represents high volume and high radiation doses [51].

One of our aims was to investigate how well the reduction in services corresponds to the recommendations of the Norwegian Choosing Wisely Campaign [28]. Our findings suggest that the practical priority setting only partly corresponded to the campaign. Most examinations were initially reduced from one to two third of the normal level but increased again in period 2 and 3. This could indicate that recommendations from the Norwegian Choosing Wisely Campaign were not followed when opening in period 3. However, the Norwegian version of the campaign include only six specific examinations and indications. Our findings suggest that several radiological examinations have potential to be low value. This corresponds to international literature where more examinations are identified by the extended list of low-value radiology [5].

It is also important to notice that the burden of disease may be different during the studied periods as there were less activity during the close-down, e.g., fewer accidents. Moreover, population studies from Norway indicates that there were fewer strokes and infarctions during the start of the pandemic [52, 53].

The data describe the practical priority setting in radiology, which is interesting in itself. However, to use the data to identify candidates for low value care, we must assume that the practical priority setting roughly follows appropriateness criteria [4, 16] and the stated priority setting principles [54]. This means that the first outpatient examinations to start up and having the highest volume after the close-down would be those of higher value than those who stay low for longer. If not, that would mean that there was no systematic priority setting, e.g., due to high pressure on the services. However, the activity at the radiological departments has been low during lock-down and there are no indications of reduced radiological capacity due to illness among radiologists or radiographers after the lock-down period [55]. While there may be some local reticence of patients to attend imaging facilities for examinations during the first part of the epidemic, no changes in “no-show” rates are reported. Moreover, if patients had been scared from coming, they would most likely not show up for issues of less importance to them.

Additionally, radiological services were affected by the reduction in other outpatient services (referring patients to radiology) and in the number of elective surgeries. While this would reduce the number of examinations with explicit prioritization at the radiology department, the reduction may be due to overall priority setting.

In this paper, we provide a methodology to investigate changes to the health services during the pandemic to identify areas for further research. The bar of 50% change and 100 examinations per month is quite high. Many low-value services may be ignored by this approach, such as low-value low-volume interventional procedures. However, as there are fewer interventional procedures for the out-patient group than in the in-patient group, the loss may not be significant. Moreover, there may be many reasons for practice change, and we wanted to study the major changes.

There are also some limitations due to coding practices, which may vary. For example, there are codes for lower extremities and for foot and ankle. Furthermore, one code may be used for several clinical indications and could therefore represent both high and low value examinations. As pointed out, targeted investigation must be conducted to specify and mapping low value examinations. However, the objective of this study has not been to reveal variations or inconsistencies in coding practices, but only to study what is registered at face value and on a principal level.

Another limitation is the choice of study periods. Where to set the limits between periods is not given by nature. It is important to notice that the burden of disease may be different during the studied periods as there were less activity during the lock-down, e.g., fewer accidents, but also fewer strokes and infarctions during the start of the pandemic [52, 53]. Adding additional study periods would provide more information on backlog and lasting effects.

However, the applied periods appear to be well chosen as the first period corresponds well with the close-down, the second with the opening somewhat, and the third with opening more [56]. This is also confirmed by reports by health authorities [51]. Figure 1 also indicates that there were very few covid-19 cases after Period 3. Moreover, the total reduction in Period 2 is 25.4% which corresponds well with other studies [29–34] and with the literature on overuse in radiological services [5, 57]. It also indicates that our threshold is well selected.

The direct influence of the examinations of patients with SARS-COV-2 is expected to be very low as there were very few cases of SARS-COV-2 in Norway [58] and very few outpatient examinations related to SARS-COV-2 as well as relatively few hospitalized patients with SARS-COV-2 that could influence the number of examinations of outpatients. However, there can be an indirect influence, e.g., cancelled elective outpatient treatments and surgery resulting in reduced pre/postoperative outpatient examinations and controls.

There are many ways to measure reduction. We have used percentage reduction compared to (the average of) the same period previous years, and to use Period 2 and 3 (and not Period 1) to identify potential low-value examinations. However, we noticed that examinations with the highest reduction rate for the various modalities are of relatively low volume examinations. Therefore, further research focusing on high volume examinations and examinations with potential high radiation dose would be welcome.

The results are specific for Norway and for outpatient radiological services. However, the results concur with other studies (on imaging and other services as referred above), and the identified examinations may be relevant for other countries, given the broad international collaboration and alignment. Moreover, this study presents a methodology to analyze the value of health services in other fields as well.

It is too early to verify that the identified examinations are of low value as the long-term effects of the reduction in 2020 are not assessable yet. Nonetheless, this study gives insights in practical priority setting and provides a specific set of potential low-value radiological examinations, and it presents a methodology for identifying them. The next step towards quality improvement is thorough analysis of the specific examinations, the corresponding clinical indications, and the assessment of the long-term effects of reduced services.

## Conclusion

In this study, we propose and demonstrate a methodology using the SARS-COV-2 pandemic as a natural intervention to investigate practical priority setting in radiological services in Norway. We found a substantial reduction of the number of outpatient examinations, indicating how specific radiological examinations are valued in practice. As such, it can help us identifying potential low value radiological services. The imaging examinations with the most continuing reduction during the initial phases of the pandemic in 2020 was Abdominal KUB view, CT Enterography, MRI Pelvis and lower limb, Throat and neck ultrasound, MRI Thoracic spine, MRI sacroiliac joints, Liver, gall bladder and pancreatic ultrasound, Axillary ultrasound and MRI of the face. While the study confirmed a reduction in some examinations identified by the Norwegian and USA Choosing Wisely Campaign there was no clear pattern that indicated reduction in identified low-value examinations found in these guidelines. Further research should focus on in-depth analyses of examination codes, clinical indications, and long-term effects to verify specific radiological examinations as low value. Nonetheless, the SARS-COV-2 pandemic provides a natural intervention for identifying potential low value services and for quality improvement.

## Data Availability

The datasets used and/or analysed during the current study available from the corresponding author on reasonable request.

## Abbreviations

DEXA: Bone density scan
KUB: Kidney, ureters and bladder
RSD: Relative Standard Deviation
RX: Conventional radiology
SD: Standard Deviation
